# Beyond human gold standards: A multimodel framework for automated abstract classification and information extraction

**DOI:** 10.1017/rsm.2025.10054

**Published:** 2025-11-17

**Authors:** Delphine S. Courvoisier, Diana Buitrago-Garcia, Clément P. Buclin, Nils Bürgisser, Michele Iudici, Denis Mongin

**Affiliations:** 1 Rheumatology, Geneva University Hospitals, Switzerland; 2 Rheumatology, University of Geneva, Switzerland; 3 Internal Medicine, Geneva University Hospitals, Switzerland

**Keywords:** classification, data extraction, evidence synthesis, large language model, meta-analysis

## Abstract

Meta-research and evidence synthesis require considerable resources. Large language models (LLMs) have emerged as promising tools to assist in these processes, yet their performance varies across models, limiting their reliability. Taking advantage of the large availability of small size (<10 billion parameters) open-source LLMs, we implemented an agreement-based framework in which a decision is taken only if at least a given number of LLMs produce the same response. The decision is otherwise withheld. This approach was tested on 1020 abstracts of randomized controlled trials in rheumatology, using 2 classic literature review tasks: (1) classifying each intervention as drug or nondrug based on text interpretation and (2) extracting the total number of randomized patients, a task that sometimes required calculations. Re-examining abstracts where at least 4 LLMs disagreed with the human gold standard (dual review with adjudication) allowed constructing an improved gold standard. Compared to a human gold standard and single large LLMs (>70 billion parameters), our framework demonstrated robust performance: several model combinations achieved accuracies above 95% exceeding the human gold standard on at least 85% of abstracts (e.g., 3 of 5 models, 4 of 6 models, or 5 of 7 models). Performance variability across individual models was not an issue, as low-performing models contributed fewer accepted decisions. This agreement-based framework offers a scalable solution that can replace human reviewers for most abstracts, reserving human expertise for more complex cases. Such frameworks could significantly reduce the manual burden in systematic reviews while maintaining high accuracy and reproducibility.

## Highlights

**What is already known?**

- Use of LLM for research synthesis is promising for abstract classification and data extraction.
- However, performance varies considerably between models.
- A key limitation is the tendency of LLMs to hallucinate or to fail to recognize when they do not know the answer, which can hinder achieving the sensitivity required for research synthesis.

**What is new?**

- An agreement-based approach using multiple open-source small LLMs (<10B parameters) achieved >95% accuracy in classifying drug versus nondrug interventions and extracting sample sizes from article abstracts.
- The method demonstrated robust performance even when individual LLMs varied in accuracy, as the agreement strategy effectively filtered out errors.
- The study showcased that a fixed decision threshold can balance accuracy and coverage, ensuring high precision while flagging uncertain cases for human review.

**Potential impact for RSM readers**

- Our proposed framework ensures reliable use of AI screening for classification or data extraction tasks, replacing data extraction by human for most abstracts.
- Our study encourages the clinical research community to consider open-source, locally deployable AI tools as viable alternatives to proprietary models, addressing ethical and access concerns.

## 1 Introduction

Meta-research and evidence synthesis research compile and analyze evidence from primary studies relevant to clinical fields, public health, social sciences, and other areas of knowledge.^1^^,^ ^2^ Due to the exponential increase in the number of scientific publications,^3^^–^ ^5^ these research projects require considerable resources, especially in time and human effort.^6^ One of the main tasks is the selection of studies based on their abstract and title. Machine learning has recently gained attention as a potential tool to reduce workload and enhance efficiency when performing such work. Classifiers such as random forest or neuronal networks were first considered to help with the title and abstract screening process in a semiautomated way.^7^ Currently, the transformer architecture and the subsequent era of large language models (LLMs) are investigated to help reduce human workload in the screening phase. A first approach has been to train small LLMs on specific classifying tasks,^8^ which produced promising results but had the disadvantage of being very specific to the task and the training dataset. The fast improvement of LLMs has now produced highly versatile algorithms able to perform various tasks, including text completion, translation, summarization, and answering questions.^9^ LLMs have shown abilities close to or above human level in various areas such as passing exams,^10^ assessing trends and topics presented in academic meetings,^11^ developing prediction models in neuroscience,^12^ diagnostic reasoning,^13^ or critically inspecting research papers.^14^

In evidence synthesis, LLMs have shown promising results for abstract classification screening tasks,^15^^–^ ^17^ or data extraction from published studies,^18^ yet without reaching the performance thresholds needed for fully automated tasks so far.^19^ Furthermore, the use of LLMs for such tasks suffers from several limitations. First, their performance vary with the specific model used and the task tested, raising concerns about generalizability and reproducibility of the results.^20^^–^ ^23^ Second, regarding reproducibility, information on retraining of the models is often lacking, which may lead to unknown changes in performance. Third, larger models tend to give wrong answers more confidently,^24^ raising concerns of potential harm for evidence synthesis. Finally, most of the studies so far rely on proprietary cloud-based LLMs, leading to ethical questions about data ownership and access to proprietary or sensitive information when using full texts.^25^

Taking advantage of the large availability of medium- (between 10 and 40 billion parameters) or small-sized (<10 billion parameters) open-source LLMs,^9^ we implemented an agreement-based approach in which a decision is taken only if at least a given number of LLMs produce the same response (decision threshold) or the decision is withheld. This approach was tested on abstracts of randomized controlled trials in rheumatology published from 2009 to 2022,^26^ using 8 different LLMs. The LLMs performed 2 classic literature review tasks: (1) classifying each intervention as drug or nondrug based on text interpretation and (2) extracting the total number of randomized patients. Results were compared to state-of-the-art LLMs as well as conventional human gold standard.^27^ A “platinum standard” was obtained by humans thoroughly rechecking all abstracts where at least 4 LLMs had all agreed to a different answer compared to the human gold standard. LLM decision results and the human gold standard were then compared to this new platinum standard.

## 2 Methods

### 2.1 Abstracts analyzed

The included abstracts are primary reports of RCTs in rheumatology published between 2009 and 2022, used in a previous study.^26^ They were used for 2 tasks:

- Binary classification where the abstracts were classified based on interventions testing a drug or not (nondrug).
- Extraction of the number of randomized patients in the RCTs, or reporting as “missing” if the information was not present.

For the second task, humans also reported if the number was given as total or by arm, thus requiring calculations.

### 2.2 List of LLMs considered

In all, 8 openly available LLMs of less than 10 billion parameters were considered:

- Llama 3 8B from Meta AI^28^
- Ministral 8B from Mistral^29^
- Qwen 2.5 7B from Alibaba Qwen^30^
- Yi 1.5 9B from 01.ai^31^
- Gemma 2 9B from Google^32^
- Deepseek 7B from Deepseek AI^33^
- Phi 3 small (7B) from Microsoft^34^
- Aya expanse 8B from Cohere For AI^35^

We used the instruct version of these models, which were downloaded from Huggingface and used in python through the transformer library.

Two state-of-the-art models were considered:

- Llama 3.3 70B, from Meta AI^36^
- Qwen 2.5 72B, from Alibaba Qwen^37^

### 2.3 The gold standard

Both tasks were performed independently by 2 human reviewers (health care professionals: statisticians and epidemiologists). In case of disagreement, adjudication was performed by a third reviewer, yielding the human gold standard.

### 2.4 The platinum standard

When more than 4 LLMs provided a concordant answer that was different from the human gold standard, the human gold standard was revised by a fourth reviewer, who had access to the LLM justifications. The subsequent reference, named the platinum standard, was considered as the reference standard in all future analyses.

### 2.5 LLM inference

**Figure 1:**
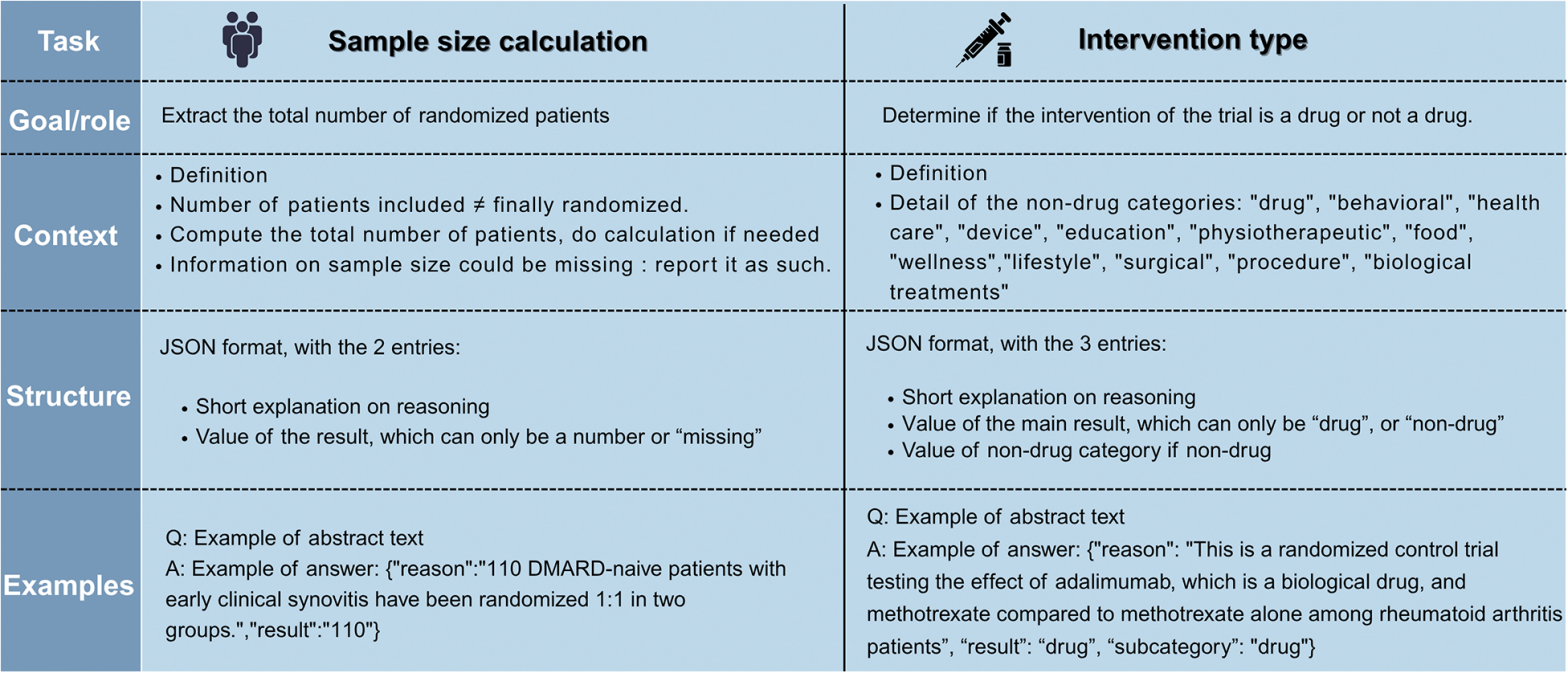
Structure of the prompt for the 2 classification tasks.


Let 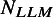 be the number of LLMs included in the task and 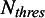 the agreement threshold. Our framework considered that an answer was valid if provided by at least 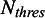 LLMs. If this number of LLMs agreeing was not reached, then the task was considered as nonanswered for the given abstract (Figure 1). For the classification task, agreement was reached if 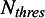 LLMs classified the abstract as being of the same category (drug or nondrug). For instance, if 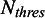 is 5 and 7 LLMs considered, and 5 LLMs or more classify the abstract as nondrug, then agreement is considered reached. If only 4 LLMs classify the abstract as nondrug, then agreement is not reached, and the task is considered as nonanswered. For the sample size task, agreement was reached if 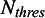 LLMs reported the exact same number.

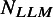
 was varied between 3 and 7, and all combinations among the 8 LLMs were tested, leading to 56, 70, 56, 28, and 8 LLM combinations, respectively. 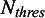 varied between half 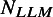 rounded to the lowest integer and 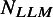 .

For example, considering the sample size task, fixing 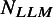 as 6 and 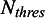 as 4, each abstract would be evaluated by a selection of 6 specific LLMs among 8, and the procedure would provide a classification only if 4 models provided the exact same sample size (perfect agreement). This procedure would then be done for each possible draw of 6 LLMs among 8.

### 2.6 Prompt and parameters

All LLMs were given the same prompt and parameters. We set a random sampling with cumulative probability and the most likely next word sampling (top_p and top_k), considering top_k = 40 and top_p = 0.95 and a temperature of 0.4. A sensitivity analysis varied the top_p, top_k, and temperature parameters, using the Qwen 2.5 7B model. We performed one inference without sampling (greedy search), and, with sampling, the top_p parameter was varied from 0.95 to 0.80, top_k from 40 to 20, and temperature between 0.2, 0.4, and 0.8.

The prompt was structured as follows (Figure 1):

- Definition of LLM role and objective
- Definition of the task and context (what is a drug and nondrug intervention, what is the number of randomized patients)
- Definition of the output structure in JSON, with a first entry for an explanation, following the idea of chain of thought structure^38^
- few shots prompting (5 examples)

Prompts, python code, inference results, computer configuration, and R code for the analysis are available at https://gitlab.unige.ch/trial_integrity/llm_majority_public.

### 2.7 Statistical analysis

The main performance metric for both tasks is the accuracy, defined as the number of correct answers compared to the platinum reference divided by the total number of queries. The accuracy between the combination of LLMs and the human gold standard was compared using t-test.

For the classification task, recall (also called sensitivity, defined as the proportion of the label properly guessed) and F1 scores, defined as the harmonic mean of the precision and recall (or positive predictive value) are also provided for both categories (drug and nondrug).

All analyses and figures were performed in R 4.4.2.^39^

## 3 Results

The 1020 included abstracts were published in more than 20 journals, the most frequent being *BMC Musculoskeletal Disorders* (17.9%), followed by *Annals of the Rheumatic Diseases* (14.3%). Wide scope medical journals also contributed (e.g., Lancet: 5.3%; JAMA: 2.9%). The conditions studied were diverse, the most frequent being osteoarthritis (20.9%), and rheumatoid arthritis (20.2%).

LLMs were able to provide a readable JSON output on almost all abstracts, although one model (Deepseek 2.5) encountered more issues, especially for the drug/nondrug classification (Table 1, column format issue). Thus, the LLM decision could sometimes be missing, because there were not enough models’ responses to reach the decision threshold. Both tasks required approximately 2 to 5 seconds per abstract and between 16 and 23 GB of Video Random Access Memory (Vram) for all small LLMs, while it took around 145 GB of Vram and 9 seconds per abstract for the 2 larger models (Table 1).

**Table 1:** Accuracy of individual models, single human reviewer and human gold standard, for 2 tasks: The classification of the intervention (drug versus nondrug) and the number of randomized patients

Task evaluator	Classification of Intervention drug/nondrug				Number of patients			
	Accuracy (%)	Format issue	Time (s)	Vram (Go)	Accuracy (%)	Format issue	Time (s)	Vram (Go)
Human gold	96.30				98.30			
Reviewer	89.38				94.55			
Qwen25 70b	94.31	0	9 [9–10]	144.7 [144.7–144.8]	96.59	0	9 [8–10]	148.7 [148.6–148.7]
Llama33 70b	94.69	0	10 [9–11]	149.1 [149–149.2]	96.97	4	9 [8–11]	144.1 [144.1–144.2]
Qwen 2.5 7b	93.45	0	3 [2–3]	16.4 [16.4–16.4]	94.98	0	2 [2–2]	16.3 [16.2–16.3]
Phi3 small	90.61	1	5 [4–5]	24.5 [24.3–24.6]	91.29	0	3 [3–4]	23.6 [23.6–23.9]
Ministral 8b	89.75	0	3 [2–3]	17.3 [17.3–17.4]	96.02	0	2 [2–3]	17.2 [17.2–17.2]
Llama3 8b	90.04	0	3 [2–3]	17.3 [17.3–17.4]	95.55	4	2 [2–3]	17 [17.0–17.2]
Granite30 8b	82.54	13	5 [4–6]	19.8 [19.8–19.9]	72.25	7	3 [3–4]	19.1 [19.1–19.2]
Gemma2 9b	92.50	0	5 [4–5]	21.9 [21.8–21.9]	96.78	0	4 [3–5]	21.2 [21.2–21.3]
Deepseek 7b	64.23	104	5 [4–5]	16.7 [16.7–16.8]	77.65	20	4 [3–5]	16.2 [16.1–16.3]
Aya 8b	92.88	0	2 [2–3]	17.6 [17.6–17.6]	89.49	0	3 [3–3]	17.2 [17.1–17.2]

**Figure 2:**
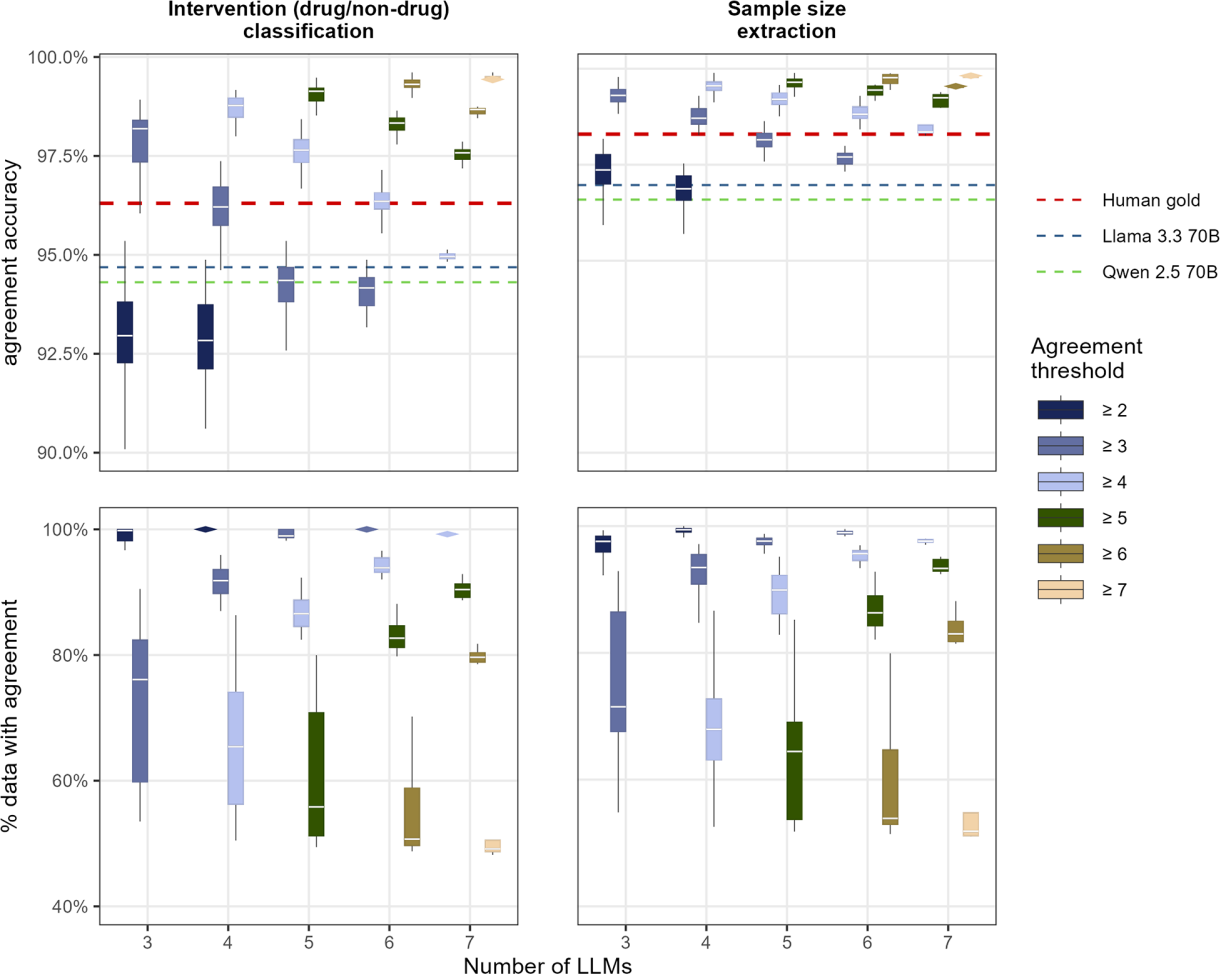
Considering all combinations of LLMs, for a given number of LLM (*x*-axis) and a given agreement threshold (filling color), the figure presents the resulting accuracy of the agreement (top panels) and the corresponding percentage of data with an agreement result (bottom panels) for both tasks: classification of the intervention between drug and nondrug (left panels), or extraction of the number of randomized patients (right panels). When the boxplots were too thin, they were replaced by a diamond-like shape.

### 3.1 Improving the gold standard

The human gold standard (2 human reviewers and a human adjudicator) classified 447 (43.8%) abstracts as containing a drug as an intervention. In 88 cases, at least 4 LLMs classified the abstracts differently than the humans, and 38 (43.2%) were subsequently reclassified in favor of the LLM agreement decision. All but one were reclassified from a drug to a nondrug intervention RCT, leading to the final platinum standard of classifying 411 abstracts as containing a drug intervention: 447 abstracts initially classified as drug intervention, minus 37 reclassified as nondrug intervention plus one reclassified as drug intervention. This corresponds to an accuracy of 96.3% (982 correctly classified and 38 misclassified out of 1020) for the human gold standard. Similarly, for the extraction of the number of randomized patients, the agreement of at least 4 LLMs yielded different results than the human gold standard for 45 abstracts out of which 18 (40%) were subsequently changed in favor of the LLM agreement. Thus, the human gold standard had an accuracy of 98.3%. The cases where the agreement of 4 LLMs differed from the human gold standard were in a vast majority situation for which the 2 human reviewers disagreed, and adjudication was needed.

### 3.2 Performance

Overall, the human gold standard and most of the LLMs had better accuracy to extract sample size compared to drug classification (Table 1). Qwen 2.5 7b, Phi3 small, Ministral, Llama 3 8b, and gemma2 9b had a similar accuracy compared to single human reviewers for both tasks. Aya 8B, deepseek 7B, and granite 3.0 8B tended to be less accurate than human reviewers, while large models (70 billion parameters) were better than a single human reviewer for both tasks but still slightly less accurate than the human gold standard. Both types of single raters had a lower accuracy compared to the human gold standard, with most ranging close to 90% for the drug/ nondrug classification task, and close to 94% for the sample size task (Table 1).

Figure 2 presents the accuracy (top panel) and the percentage of abstracts with an agreement result (bottom panel) of the agreement of LLMs, for a given number of LLMs (*x*-axis) and a given agreement threshold (filling color). Combining the LLMs increased the accuracy of the agreement result, with the accuracy steadily increasing from below the human gold to nearly 100% when increasing the decision threshold. This increase in agreement was at the cost of a decreased percentage of abstracts obtaining an agreement. In other words, the number of abstracts having enough LLMs agreeing to reach an agreement decreased when increasing the decision threshold. For instance, when selecting combinations of 3 LLMs out of the 8 available, 2 agreement thresholds can be applied: either at least 2 models must agree, or all 3 must agree. The resulting accuracy distributions for these thresholds are shown in the first and second boxplots of the upper left panel in Figure 2, with median accuracies of 92.9% and 98.2%, respectively (see Supplementary Table S1 for the median and interquartile range of all accuracies). The corresponding proportions of abstracts for which agreement was reached under these thresholds are presented in the first and second boxplots of the lower left panel, with median agreement rates of 99.8% and 76.1%, respectively (Supplementary Table S2). Other performance metrics (precision, recall, and F1) showed similar patterns of results for all ratings (see Supplementary Tables S1–S14 for all performance metrics, including precision, recall or F1 measurements).

Considering together the number of abstracts receiving an LLM agreement rating and the accuracy, several combinations of the models reached a performance equal to or above the human gold standard accuracy, while still evaluating at least 85% of the abstracts: agreement of at least 3 models out of 4, agreement of at least 4 models out of 5, agreement of at least 4 models out of 6, or agreement of at least 5 models out of 7 (Figure 2). In more detail, an agreement of at least 4 models out of 5 was achieved for 86.6% (IQR: [84.5; 88.8]) and 89.9% (IQR: [86.2; 92.2]) of the abstracts for the intervention classification task and the sample size extraction task, respectively (Figure 2, bottom panels, light blue boxplot, for *x*-axis = 5, and Supplementary Tables 2 and 10, line for N model = 5 and Threshold = 4). This resulted in the correct result (that is, the platinum standard) of a median of 97.8% (IQR: [97.5; 98.1]) and 99.2% (IQR: [99.1; 99.4]) of the time (Figure 2, top panels, light blue boxplot, for *x*-axis = 5, and Supplementary Tables 1 and 9, line for N model = 5 and Threshold = 4). To achieve an optimal balance between agreement and accuracy, a more parsimonious approach would be to use the agreement of at least 3 out of 4 LLMs, a situation where an agreement is reached for 91.8% (IQR: [89.8; 93.6]) and 93.5% (IQR: [90.8; 95.5]) of the abstracts (Figure 2, bottom panels, light blue boxplot, for x-axis = 4, and Supplementary Tables 2 and 10, line for N model = 4 and Threshold = 3), yielding a median accuracy of 96.2% (IQR: [95.7; 96.7]) and 98.7% (IQR: [98.6; 99.0]), respectively (Figure 2, top panels, light blue boxplot, for *x*-axis = 4, and Supplementary Tables 1 and 9, line for N model = 4 and Threshold = 3).

**Figure 3:**
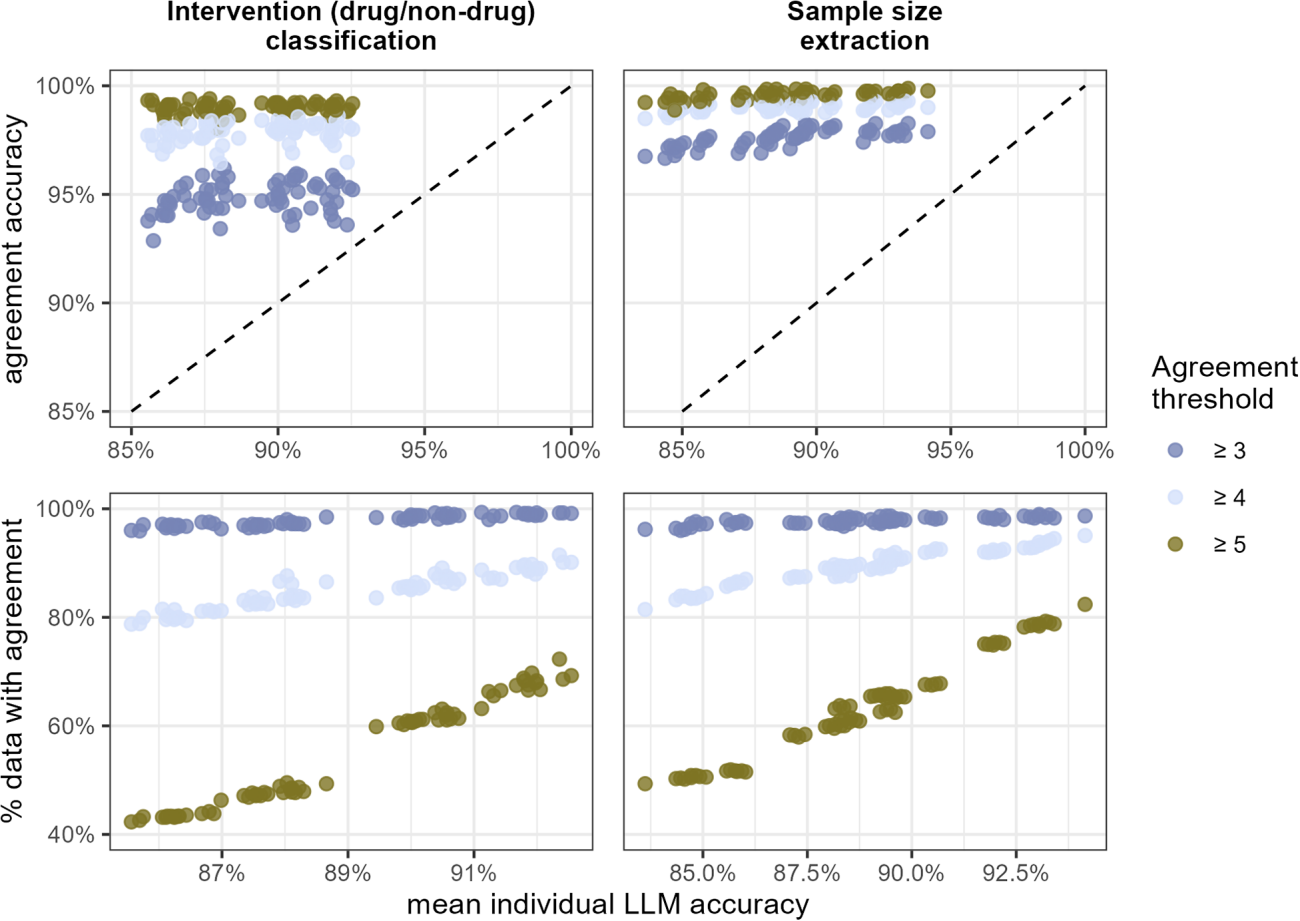
Considering all combinations of 5 LLMs (one point per combination, 56 points in total) for both tasks: classification of the intervention (left panels), or extraction of the number of randomized patients (right panels): Top panels: comparison between the accuracy of the agreement results (vertical axis) and the mean accuracy of the individual LLMs. Dashed lines indicate the same values for agreement accuracy and individual accuracy. Bottom panels: comparison between the percentage of data with an agreement (vertical axis) and the mean accuracy of the individual LLMs. Mean accuracy is computed by taking the average of the accuracy of each LLM model for a given task. As we considered 8 LLMs, when studying the use of 5 LLMS, for example, we considered all possible combinations of LLMs within the 8 available, with each combination contributing one point in the figure (56 possible LLM combinations in total).

The choice of the individual LLM had almost no influence on the decision accuracy (Supplementary Figures 1 and 2), primarily because combinations involving lower-performing models were less likely to reach agreement. This pattern is shown in Figure 3, which examines combinations of 5 LLMs in detail. The proportion of abstracts for which agreement was reached declined almost linearly with the individual accuracy of the selected LLMs, with steeper declines observed at higher decision thresholds. For instance, for the intervention classification task, the percentage of abstracts reaching agreement with 4 models (Figure 3, light blue dots) went from 90% to 80% when comparing the combination of the most accurate individual LLMs (on the right side of the graph) compared to the less accurate ones (on the left side of the graph), whereas the agreement of 5 LLMs changed from 70% to almost 40% (Figure 3, left panels).

Changes of the models’ parameters did not affect the LLM individual performances. Indeed, the accuracy of Qwen 2.5 7B varied from 92.4% to 93.4% when changing parameters for the intervention classification task, and from 92.8% to 96.1% for the extraction of randomized participants, the best accuracy being obtained with the highest temperature (data not shown).

### 3.3 Subgroup analysis

The performance of the LLMs in detecting the 22 abstracts (out of 1020) that did not report any sample size was generally good, with a precision >90% when at least 3 LLMs classified the sample size as “missing,” while the recall remained at 90% (Supplementary Tables S9–S11).

For the sample size extraction task, the performance of the individual human reviewer was 23 percent points lower when calculating the sample size was required, such as abstracts presenting treatment groups separately, necessitating the summation of both arms to determine the total. This loss of individual performance also impacted the human gold standard. Although the adjudication corrected most of the initial human mistakes, situations where both reviewers did the same mistake or where the human adjudicator was wrong still left a 3 percent point difference between abstracts that did not require computation (99.9%) and abstracts that required computation (97%). In contrast, individual LLMs made less calculation errors when computing the sample size, with a difference in the accuracy between 3 and 6 percent point. As a result, the agreement obtained had a difference in accuracy of less than 1 percent point between situations that required a calculation and those that did not require it (Supplementary Tables S12 and S13).

## 4 Discussion

We analyzed abstracts from published RCTs in rheumatology to systematically study the accuracy of an agreement-based prediction of a combination of several LLMs of size inferior to 10 billion parameters. We focused on 2 key tasks of evidence synthesis: classification of an intervention as drug or nondrug and extracting the number of randomized patients. The performance of the LLM agreement was compared to a human gold standard. This agreement-based approach has 2 advantages. First, the LLMs decision of at least 4 models can be used to improve the human gold standard. Human reviewers could re-examine the abstracts for which human and LLM decisions differed, an important advantage as it is well known that even the gold standard resulting from the 2 reviewers and an adjudicator does not provide 100% accuracy.^40^^,^ ^41^ The second advantage is that requiring an agreement between a fixed number of LLMs before considering an answer as valid yielded accuracies surpassing both the human gold standard and the state-of-the-art LLMs. The accuracy of the results increased when the threshold (that is, number of LLMs needing to agree with each other) to reach a decision was increased, although this meant achieving an agreement on fewer abstracts. Consequently, this approach remained robust, regardless of the individual accuracy of the LLMs used, as lower-performing models produced fewer agreements.

This simple yet effective method addresses the issue raised by Zhou et al.,^24^ who highlighted that LLMs are prone to confidently generate incorrect responses, especially for larger LLMs. Requiring a minimum number of LLMs to provide concordant answers before accepting a response as valid (and rejecting cases where disagreement occurs) enhanced reliability. Incorrect responses were naturally filtered out, as different LLMs trained on different datasets produced varying incorrect answers, making disagreements a strong indicator of inaccuracy. At the same time, this simple principle made it robust against the performance variation across models, potentially tackling the persistent problem of performance changes across models,^17^^,^ ^21^^,^ ^42^ the prompt used,^15^^,^ ^21^^,^ ^22^^,^ ^43^ or even across time for the same proprietary model.^44^

Several approaches using multiple LLMs to improve accuracy have been suggested in the literature. One involves an *active voting* of LLMs,^45^^,^ ^46^ where the LLMs are given other models’ answers as prompt and must collectively determine the correct answer, potentially after debate.^47^ Another approach simply takes the majority answer from multiple LLMs, which have been experimented in some studies^17^^,^ ^48^ and have shown an improvement in their performance. To our knowledge, only one study used a fixed decision threshold to accept or reject the answer proposed by various LLMs,^49^ with performance improvement in line with our study. It was, however, limited by the use of only 2 proprietary LLMs (GPT-4 and Claude 3).

Although this agreement-based approach improves accuracy, it implies that 10%–15% of the abstracts will not obtain a decision, due to the model’s conservative approach in cases where not enough models agree. To address these unclassified abstracts, 2 primary strategies can be employed. The first involves relying only on the human gold standard for these abstracts. The amount of time saved by using LLMs could potentially be invested into adding more human reviewers on these more difficult abstracts. The second approach entails using a single, larger model in conjunction with the human reviewer, allowing for more nuanced decision-making while maintaining efficiency. Both methods offer a means of resolving uncertainty while balancing precision and effort.

Our proposed method leverages the availability of numerous open-source LLMs, each with less than 10 billion parameters, requiring less than 20GB of VRam when performing inference, and able to run on consumer grade hardware. Many of these models already achieve performance levels comparable to human reviewers for the 2 tasks examined in this study, support large-context inputs, can be run locally, and are freely accessible.

This study has several strengths. First, our agreement-based approach uses openly available small LLMs, which only take a few seconds to analyze an abstract, corresponding to less than 7 hours to process all abstracts by 5 different models. Second, the factorial design considering all possible combinations of models among 8 LLMs provided a clear picture of the variability in performance due to the choice of models. Finally, the use of openly available data and code ensures adequate reproducibility.

The main limitation concerns the generalizability of the findings of this study. Though we considered 2 different tasks, and both had strikingly similar excellent accuracy, performance could be lower for other tasks. Similarly, performance could differ with a different sample of abstracts. Finally, accuracy of decisions based on full-text articles remains to be assessed.

## 5 Conclusion

The agreement-based approach to 2 tasks necessary for evidence synthesis yielded excellent accuracy (>95%) on at least 85% of abstracts, while taking less than 7 hours if used with 5 models. Although these promising results should be confirmed on other tasks and datasets, they pave the way to greatly facilitating evidence synthesis, as they could allow to replace human reviewers by artificial intelligence for most abstracts.

## Supporting information

Courvoisier et al. supplementary material Courvoisier et al. supplementary material

## Data Availability

All code and data can be openly found at the following registry: https://gitlab.unige.ch/trial_integrity/llm_majority_public, and at the Zenodo repository https://doi.org/10.5281/zenodo.15829040.^50^

## Supplementary material

To view supplementary material for this article, please visit http://doi.org/10.1017/rsm.2025.10054.
