# Supplementary material for: Beyond human gold standards: A multimodel framework for automated abstract classification and information extraction
Source: Res Synth Methods. 2025 Nov 17;17(2):365–77. doi: 10.1017/rsm.2025.10054 (PMC12873610; doi:10.1017/rsm.2025.10054)

## Computer configuration

All tasks using models below 10B were run on NVIDIA RTX 3090 of 25GB VRam Graphical Processing Units (GPU) on a 2.25GHz AMD EPYC 7742 computer processing unit (CPU) with 30GB of Ram.
The tasks with the two state-of-the-art models were run locally on two NVIDIA A100 of 80GB of VRam GPU, on a 2.25GHz AMD EPYC 7742 CPU with 160GB of Ram.

The time needed for a task is estimated as the mean of the time elapsed between the analysis of two abstracts and does not account for the time needed to load the LLM.

# Supplementary Tables for the drug non-drug classification task

Table S1: median [Inter Quartile Range] accuracy, as a function of the Number of LLMs models and of the agreement threshold.

| Threshold  N model | 2 | 3 | 4 | 5 | 6 | 7 |
| --- | --- | --- | --- | --- | --- | --- |
| 3 | 92.9 [92.2; 93.8] | 98.2 [97.3; 98.4] |  |  |  |  |
| 4 | 92.8 [92.1; 93.7] | 96.2 [95.7; 96.7] | 98.8 [98.5; 99] |  |  |  |
| 5 |  | 94.4 [93.8; 94.7] | 97.6 [97.3; 97.9] | 99.1 [98.9; 99.2] |  |  |
| 6 |  | 94.2 [93.7; 94.4] | 96.4 [96.2; 96.6] | 98.3 [98.2; 98.5] | 99.3 [99.2; 99.4] |  |
| 7 |  |  | 95 [94.9; 95] | 97.6 [97.4; 97.7] | 98.7 [98.6; 98.7] | 99.4 [99.4; 99.5] |

Table S2: median [Inter Quartile Range] percentage of data with an agreement, as a function of the Number of LLMs models and of the agreement threshold.

| Threshold  N model | 2 | 3 | 4 | 5 | 6 | 7 |
| --- | --- | --- | --- | --- | --- | --- |
| 3 | 99.8 [98.2; 100] | 76.1 [59.8; 82.4] |  |  |  |  |
| 4 | 100 [100; 100] | 91.8 [89.8; 93.6] | 65.4 [56.2; 74.1] |  |  |  |
| 5 |  | 99 [98.7; 100] | 86.6 [84.5; 88.8] | 55.8 [51.2; 70.8] |  |  |
| 6 |  | 100 [100; 100] | 93.9 [93.1; 95.5] | 82.7 [81.2; 84.7] | 50.7 [49.7; 58.8] |  |
| 7 |  |  | 99.2 [99.2; 99.4] | 90.4 [89.2; 91.3] | 79.6 [78.8; 80.4] | 49.1 [48.7; 50.5] |

Table S3: median [Inter Quartile Range] precision of the drug classification, as a function of the Number of LLMs models and of the agreement threshold.

| Threshold  N model | 2 | 3 | 4 | 5 | 6 | 7 |
| --- | --- | --- | --- | --- | --- | --- |
| 3 | 90.7 [88.2; 92.8] | 97.4 [95.6; 98] |  |  |  |  |
| 4 | 87.5 [85.3; 89.3] | 94.5 [93; 95.7] | 98.3 [97.5; 98.7] |  |  |  |
| 5 |  | 92 [90.5; 93.2] | 96.3 [95.4; 97.3] | 98.7 [98.3; 99] |  |  |
| 6 |  | 89.6 [88; 90.5] | 94.3 [93.5; 95.6] | 97.4 [96.9; 97.9] | 99 [98.6; 99.1] |  |
| 7 |  |  | 92.3 [91.4; 92.8] | 96.1 [95.7; 96.6] | 98 [97.8; 98] | 99.2 [99; 99.4] |

Table S4: median [Inter Quartile Range] recall of the drug classification, as a function of the Number of LLMs models and of the agreement threshold.

| Threshold  N model | 2 | 3 | 4 | 5 | 6 | 7 |
| --- | --- | --- | --- | --- | --- | --- |
| 3 | 92.1 [87.5; 96.7] | 98.2 [97.1; 98.7] |  |  |  |  |
| 4 | 97.1 [95.2; 97.8] | 97 [94.6; 97.7] | 98.6 [98; 99] |  |  |  |
| 5 |  | 94.3 [91.9; 96.5] | 97.8 [97.1; 98.3] | 98.9 [98.3; 99.2] |  |  |
| 6 |  | 97.1 [96; 97.3] | 97.1 [95.8; 97.4] | 98.2 [97.7; 98.5] | 99.1 [98.8; 99.4] |  |
| 7 |  |  | 95.2 [94.3; 96.4] | 97.5 [97.1; 97.8] | 98.4 [98.3; 98.7] | 99.4 [99; 99.4] |

Table S5: median [Inter Quartile Range] F1 of the drug classification, as a function of the Number of LLMs models and of the agreement threshold.

| Threshold  N model | 2 | 3 | 4 | 5 | 6 | 7 |
| --- | --- | --- | --- | --- | --- | --- |
| 3 | 91.3 [90; 92.4] | 97.4 [96.5; 97.8] |  |  |  |  |
| 4 | 91.3 [90.4; 92.4] | 95.3 [94.5; 95.7] | 98.2 [97.8; 98.5] |  |  |  |
| 5 |  | 93 [92.3; 93.3] | 96.8 [96.5; 97.2] | 98.7 [98.4; 98.9] |  |  |
| 6 |  | 92.9 [92.4; 93.2] | 95.4 [95; 95.7] | 97.7 [97.5; 97.9] | 98.9 [98.8; 99.1] |  |
| 7 |  |  | 93.7 [93.6; 93.8] | 96.9 [96.6; 96.9] | 98.1 [98; 98.2] | 99.1 [99.1; 99.3] |

Table S6: median [Inter Quartile Range] precision of the non-drug classification, as a function of the Number of LLMs models and of the agreement threshold.

| Threshold  N model | 2 | 3 | 4 | 5 | 6 | 7 |
| --- | --- | --- | --- | --- | --- | --- |
| 3 | 94.7 [92.1; 97.7] | 99 [98.5; 99.2] |  |  |  |  |
| 4 | 98 [96.8; 98.4] | 98.1 [96.8; 98.4] | 99.2 [99.1; 99.4] |  |  |  |
| 5 |  | 96.2 [94.8; 97.6] | 98.6 [98.3; 98.9] | 99.4 [99.2; 99.6] |  |  |
| 6 |  | 98 [97.3; 98.2] | 98.1 [97.3; 98.3] | 98.9 [98.7; 99.1] | 99.5 [99.4; 99.7] |  |
| 7 |  |  | 96.8 [96.2; 97.6] | 98.4 [98.3; 98.6] | 99.1 [99.1; 99.2] | 99.7 [99.6; 99.7] |

Table S7: median [Inter Quartile Range] recall of the non-drug classification, as a function of the Number of LLMs models and of the agreement threshold.

| Threshold  N model | 2 | 3 | 4 | 5 | 6 | 7 |
| --- | --- | --- | --- | --- | --- | --- |
| 3 | 93.9 [91.7; 95.6] | 98.4 [97.2; 99.1] |  |  |  |  |
| 4 | 91.1 [89.1; 92.6] | 96.5 [95.2; 97.5] | 99.1 [98.5; 99.3] |  |  |  |
| 5 |  | 94.7 [93.3; 95.7] | 97.7 [97.3; 98.4] | 99.2 [99.1; 99.4] |  |  |
| 6 |  | 92.7 [91.4; 93.5] | 96.2 [95.5; 97.2] | 98.5 [98.1; 98.8] | 99.4 [99.2; 99.6] |  |
| 7 |  |  | 94.8 [94.1; 95.2] | 97.6 [97.2; 97.8] | 98.9 [98.6; 98.9] | 99.7 [99.4; 99.7] |

Table S8: median [Inter Quartile Range] F1 of the non-drug classification, as a function of the Number of LLMs models and of the agreement threshold.

| Threshold  N model | 2 | 3 | 4 | 5 | 6 | 7 |
| --- | --- | --- | --- | --- | --- | --- |
| 3 | 94.2 [93.5; 94.8] | 98.6 [98; 98.8] |  |  |  |  |
| 4 | 93.9 [93.2; 94.7] | 96.9 [96.5; 97.3] | 99.1 [98.8; 99.2] |  |  |  |
| 5 |  | 95.3 [94.8; 95.6] | 98.1 [97.9; 98.3] | 99.3 [99.2; 99.4] |  |  |
| 6 |  | 95.1 [94.7; 95.4] | 97 [96.8; 97.2] | 98.7 [98.5; 98.8] | 99.5 [99.4; 99.6] |  |
| 7 |  |  | 95.8 [95.8; 95.8] | 98 [97.9; 98.1] | 99 [98.9; 99] | 99.6 [99.6; 99.6] |

Supplementary figure 1: Top panels: comparison between the accuracy of the agreement results (vertical axis) and the mean accuracy of the individual LLMs. Bottom panels: comparison between the percentage of data with an agreement (vertical axis) and the mean accuracy of the individual LLMs.


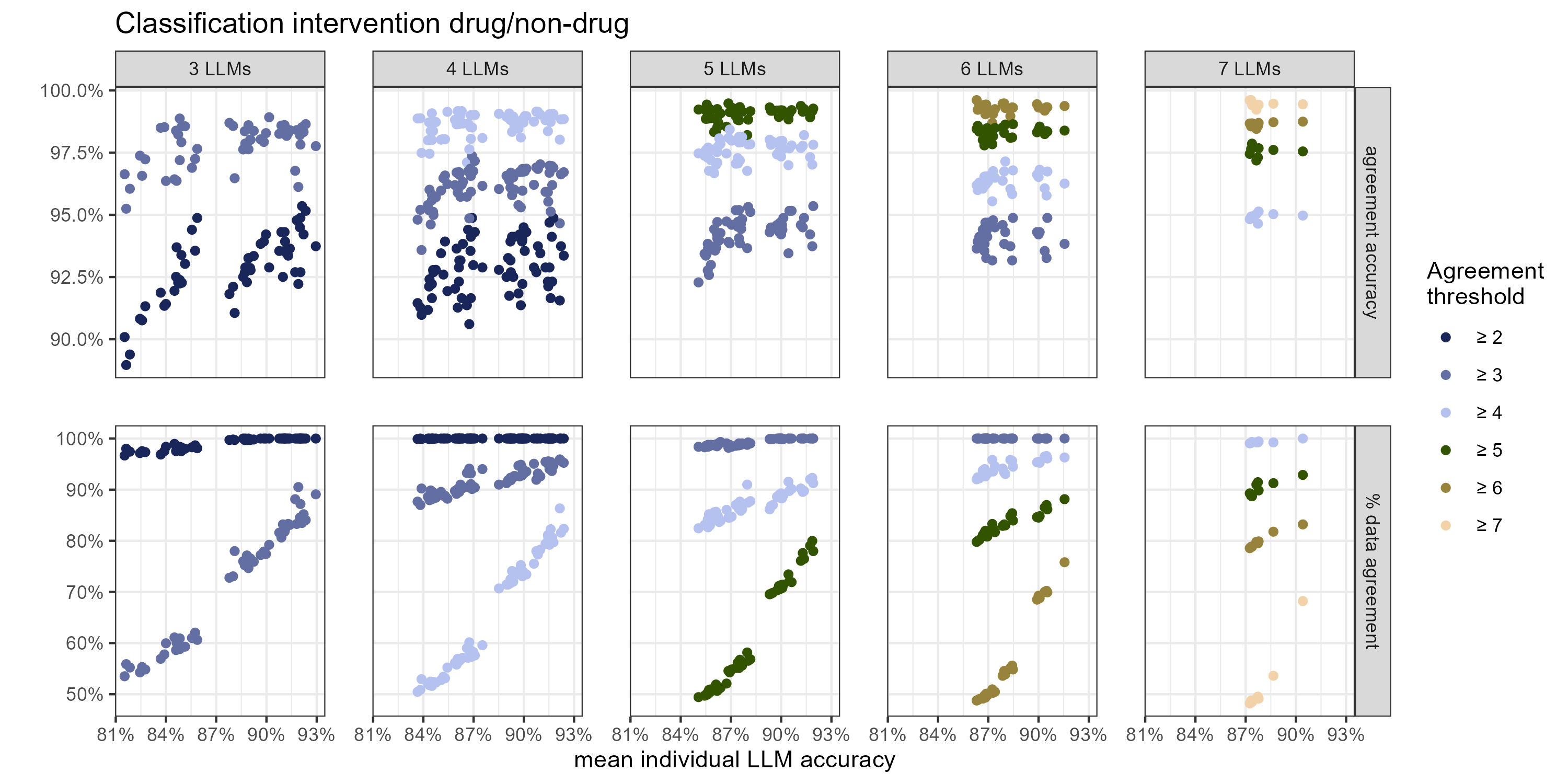


Note: mean accuracy is computed by taking the average of the accuracy of each LLM model. As we considered 8 LLMs, when studying the use of 5 LLM for example, we considered all possible combinations of LLM within the 8 available, with each combination contributing one point in the figure.

# Supplementary Tables for the extraction of randomized participants number task

Table S9: median [Inter Quartile Range] accuracy, as a function of the Number of LLMs models and of the agreement threshold.

| Threshold  N model | 2 | 3 | 4 | 5 | 6 | 7 |
| --- | --- | --- | --- | --- | --- | --- |
| 3 | 97.4 [97; 97.8] | 99.3 [99.1; 99.5] |  |  |  |  |
| 4 | 96.9 [96.6; 97.2] | 98.7 [98.6; 99] | 99.6 [99.4; 99.7] |  |  |  |
| 5 |  | 98.1 [98; 98.3] | 99.2 [99.1; 99.4] | 99.6 [99.5; 99.7] |  |  |
| 6 |  | 97.7 [97.5; 97.8] | 98.8 [98.7; 99] | 99.4 [99.3; 99.6] | 99.8 [99.6; 99.8] |  |
| 7 |  |  | 98.4 [98.3; 98.5] | 99.2 [99; 99.3] | 99.5 [99.5; 99.6] | 99.8 [99.8; 99.8] |

Table S10: median [Inter Quartile Range] percentage of data with an agreement, as a function of the Number of LLMs models and of the agreement threshold.

| Threshold  N model | 2 | 3 | 4 | 5 | 6 | 7 |
| --- | --- | --- | --- | --- | --- | --- |
| 3 | 97.6 [95.9; 98.4] | 71.5 [67.6; 86.5] |  |  |  |  |
| 4 | 99.4 [99.1; 99.6] | 93.5 [90.8; 95.5] | 67.9 [63.1; 72.8] |  |  |  |
| 5 |  | 97.6 [97.1; 98.1] | 89.9 [86.2; 92.2] | 64.4 [53.7; 69.1] |  |  |
| 6 |  | 99 [98.7; 99.2] | 95.6 [94.6; 96.1] | 86.3 [84.3; 89] | 53.9 [52.9; 64.7] |  |
| 7 |  |  | 97.6 [97.4; 97.9] | 93.3 [92.9; 94.7] | 83 [81.8; 85] | 51.8 [51.1; 54.8] |

Table S11: median [Inter Quartile Range] precision when predicting missing information, as a function of the Number of LLMs models and of the agreement threshold

| Threshold  N model | 2 | 3 | 4 | 5 | 6 | 7 |
| --- | --- | --- | --- | --- | --- | --- |
| 3 | 86.6 [66.7; 94.1] | 100 [100; 100] |  |  |  |  |
| 4 | 87.5 [80.2; 94.9] | 100 [91.6; 100] | 100 [100; 100] |  |  |  |
| 5 |  | 92.3 [85.7; 95.5] | 100 [100; 100] | 100 [100; 100] |  |  |
| 6 |  | 90.9 [84.5; 94.9] | 100 [95.1; 100] | 100 [100; 100] | 100 [100; 100] |  |
| 7 |  |  | 95.2 [93.8; 96.4] | 100 [100; 100] | 100 [100; 100] | 100 [100; 100] |

Table S12: median [Inter Quartile Range] recall when predicting missing information, as a function of the Number of LLMs models and of the agreement threshold

| Threshold  N model | 2 | 3 | 4 | 5 | 6 | 7 |
| --- | --- | --- | --- | --- | --- | --- |
| 3 | 84.2 [82.2; 91.3] | 80 [75; 93.3] |  |  |  |  |
| 4 | 84 [80; 87.9] | 87.5 [80.6; 94.6] | 75 [75; 80] |  |  |  |
| 5 |  | 87 [85.7; 90.9] | 92.3 [85.7; 94.1] | 75 [50; 80] |  |  |
| 6 |  | 87.2 [87; 87.5] | 88.9 [85.7; 94.7] | 85.7 [85.7; 92.3] | 50 [50; 76.2] |  |
| 7 |  |  | 88.5 [85.7; 90.9] | 92.8 [85.7; 94.3] | 85.7 [85.7; 87.4] | 50 [50; 56.2] |

Table S13: median [Inter Quartile Range] F1 when predicting missing information, as a function of the Number of LLMs models and of the agreement threshold

| Threshold  N model | 2 | 3 | 4 | 5 | 6 | 7 |
| --- | --- | --- | --- | --- | --- | --- |
| 3 | 84.1 [74.5; 89.4] | 87.3 [80; 94.4] |  |  |  |  |
| 4 | 85.1 [81.6; 89.4] | 90.6 [85.7; 94.4] | 85.7 [81.4; 88.9] |  |  |  |
| 5 |  | 88.9 [87; 91.3] | 93.7 [91.5; 96.6] | 85.7 [66.7; 88.9] |  |  |
| 6 |  | 88.9 [86.1; 90.2] | 92.8 [91.4; 95.2] | 92.3 [92.3; 96] | 66.7 [66.7; 86.5] |  |
| 7 |  |  | 91.6 [90.7; 93] | 96.3 [92.3; 97.1] | 92.3 [92.3; 93.2] | 66.7 [66.7; 71.4] |

Table S14: Accuracy differences when the task require calculation or not, for single LLMs or reviewers

|  | Accuracy (%) Without calculation | Accuarcy (%) With calculation | Accuracy difference (percent point) |
| --- | --- | --- | --- |
| Human Gold | 99.89 | 97.03 | 2.86 |
| Individual reviewer | 97.02 | 73.76 | 23.26 |
| Aya 8b | 91.77 | 86.14 | 5.63 |
| Deepseek 7b | 80.19 | 55.45 | 24.74 |
| gemma2 9b | 97.29 | 94.06 | 3.23 |
| granite30 8b | 71.75 | 76.24 | -4.49 |
| llama3 8b | 96.43 | 93.07 | 3.36 |
| Ministral 8b | 97.29 | 91.09 | 6.20 |
| phi3 small | 91.88 | 85.15 | 6.73 |
| qwen25 7b | 95.89 | 89.11 | 6.78 |

Table S15: Median [IQR] Accuracy of the decision result when the task require calculation or not, as a function of the Number of LLMs models and of the decision threshold

| Model Number | Decision threshold | Median accuracy (%) Without calculation | Median accuracy (%) With calculation | Median accuracy difference (percent point) |
| --- | --- | --- | --- | --- |
| 3 | 2 | 96.67 [95.53 - 97.89] | 97.8 [97.42 - 98.03] | -1 [-1.94 - -0.23] |
| 3 | 3 | 100 [100 - 100] | 99.43 [99.3 - 99.55] | 0.48 [0.35 - 0.61] |
| 4 | 2 | 94.97 [93.88 - 95.96] | 97.4 [97.17 - 97.62] | -2.42 [-3.36 - -1.36] |
| 4 | 3 | 100 [98.82 - 100] | 98.89 [98.67 - 99.06] | 0.69 [-0.18 - 1.12] |
| 4 | 4 | 100 [100 - 100] | 99.67 [99.52 - 99.8] | 0.33 [0.17 - 0.47] |
| 5 | 3 | 98.91 [97.89 - 98.96] | 98.34 [98.21 - 98.45] | 0.36 [-0.47 - 0.8] |
| 5 | 4 | 100 [100 - 100] | 99.28 [99.17 - 99.41] | 0.7 [0.55 - 0.82] |
| 5 | 5 | 100 [100 - 100] | 99.81 [99.65 - 99.86] | 0.19 [0.14 - 0.35] |
| 6 | 3 | 97.94 [97.89 - 98.21] | 97.93 [97.72 - 98.04] | -0.02 [-0.12 - 0.36] |
| 6 | 4 | 100 [100 - 100] | 98.89 [98.78 - 99.02] | 1.01 [0.76 - 1.15] |
| 6 | 5 | 100 [100 - 100] | 99.5 [99.39 - 99.62] | 0.5 [0.38 - 0.61] |
| 6 | 6 | 100 [100 - 100] | 99.92 [99.77 - 100] | 0.08 [0 - 0.23] |
| 7 | 4 | 99.48 [98.95 - 100] | 98.51 [98.46 - 98.57] | 0.96 [0.46 - 1.46] |
| 7 | 5 | 100 [100 - 100] | 99.26 [99.1 - 99.35] | 0.74 [0.65 - 0.9] |
| 7 | 6 | 100 [100 - 100] | 99.62 [99.58 - 99.63] | 0.38 [0.37 - 0.42] |
| 7 | 7 | 100 [100 - 100] | 100 [99.95 - 100] | 0 [0 - 0.05] |

Supplementary figure 2: Top panels: comparison between the accuracy of the agreement results (vertical axis) and the mean accuracy of the individual LLMs. Bottom panels: comparison between the percentage of data with an agreement (vertical axis) and the mean accuracy of the individual LLMs.


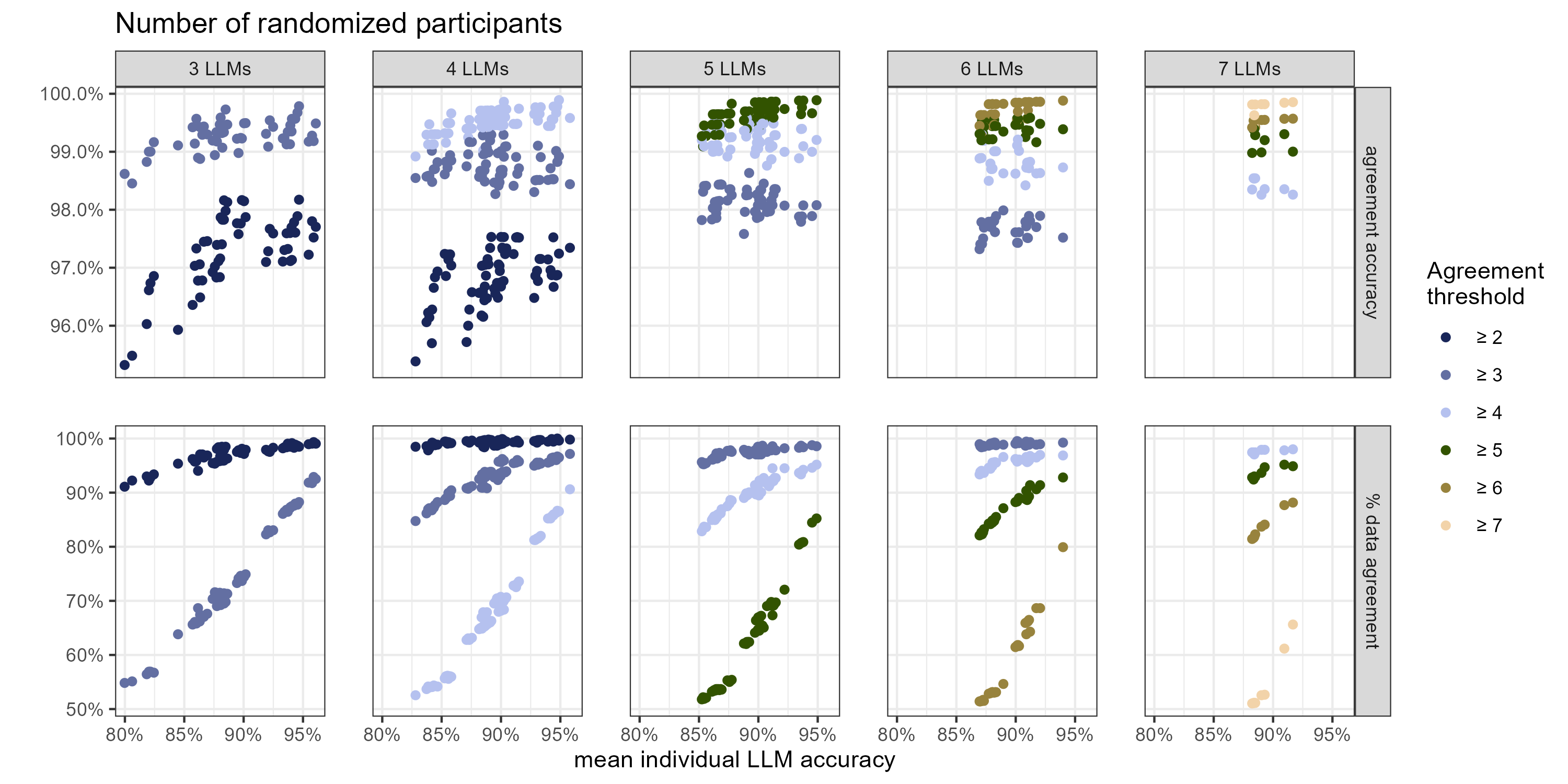

Supplement: Courvoisier et al. supplementary material [file S1759287925100549sup001.docx]
